# Automated Detection and Classification of Marine Species Vocalizations Using a YOLO‐Based Deep Learning Framework

**DOI:** 10.1002/ece3.73466

**Published:** 2026-04-15

**Authors:** Min Jun Kim, Juan Lee, Yongchae Cho, Won‐Ki Kim, Jungyong Park, Dawoon Lee, Ho Seuk Bae

**Affiliations:** ^1^ Department of Energy Systems Engineering Seoul National University Seoul Republic of Korea; ^2^ Research Institute of Energy and Resources Seoul National University Seoul Republic of Korea; ^3^ Department of Earth and Environmental Sciences Chungbuk National University Cheongju Republic of Korea; ^4^ Agency for Defense Development Changwon Republic of Korea

**Keywords:** classification, deep learning, detection, marine species vocalizations, YOLO

## Abstract

The underwater acoustic environment is highly complex, where signals from various natural and anthropogenic sources interact and overlap, making monitoring efforts very challenging. Thus, effective detection and classification mechanisms are vital, as they provide key information about marine species and help in understanding how human activities influence the overall marine environment. This study proposes a deep learning–based framework for the automatic detection and classification of marine species vocalizations, inspired by the YOLO (You Only Look Once) architecture. However, a major limitation in developing such frameworks is the limited availability of continuous, well‐annotated monitoring datasets that contain multi‐species recordings. To address this limitation, synthetic monitoring datasets were constructed by combining single‐species vocalizations to simulate realistic monitoring conditions under both non‐overlapping and overlapping scenarios. Augmentation techniques, including CutMix, were implemented to enhance dataset diversity and improve the model's robustness against signal overlap. Experimental results demonstrated that the proposed model achieves strong performance under non‐overlapping conditions and maintains stable detection and classification performance even in overlapping scenarios. These findings suggest that YOLO‐inspired architectures can achieve effective performance across various acoustic conditions. Future studies should focus on incorporating continuous, long‐term field recordings to further improve detection and classification reliability.

## Graphical abstract

This study proposes a YOLO‐inspired deep learning framework to automatically detect and classify marine species vocalizations, using a synthetic dataset to simulate realistic overlapping and non‐overlapping conditions. The model demonstrated strong performance and high precision, establishing a baseline for implementing real‐time passive acoustic monitoring systems in complex marine soundscapes.

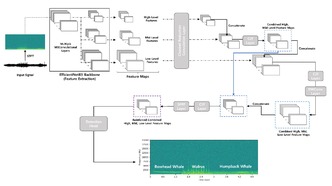

## 1 Introduction

Marine species vocalizations provide valuable information for tracking species distribution and identification, offering important insights for ecological research. However, the complexity of the underwater acoustic environment, combined with increasing anthropogenic noise, poses significant challenges to these monitoring efforts (Mellinger et al. 2007; Merchant et al. 2016). In addition, the wide diversity of vocalization types among marine species further complicates the process (Van Opzeeland et al. 2010). Therefore, effective monitoring systems are crucial, as they offer valuable insights into population dynamics and behavioral ecology while helping to assess the impacts of human activities on marine ecosystems.

Many studies have sought to develop methods for detecting and classifying marine species' vocalization to effectively monitor the marine acoustic environment. Traditional approaches have relied on manual analysis of raw recordings and conventional signal processing techniques, such as threshold‐based energy detection and spectral analysis. Specifically, Erbe and King (2008) employed information entropy, a measure of a signal's information content, to detect vocalizations based on the principle that animal sounds differ from background noise in terms of their spectral energy distribution. Although this approach is effective in distinguishing signals from background noise, it becomes impractical for large‐scale datasets and performs poorly in acoustically complex environments.

To address these limitations, recent studies have applied artificial intelligence techniques, as they offer powerful alternatives to conventional approaches by efficiently processing and analyzing large volumes of data. For instance, statistical machine learning methods have been introduced and applied in various studies. Specifically, Jarvis et al. (2008) used support vector machines to automatically classify beaked whale calls, Brown and Smaragdis (2009) applied Gaussian mixture models and hidden Markov models to categorize different killer whale vocalizations, and Amorim et al. (2022) employed random forest algorithms to distinguish between dolphin species based on their whistle sounds. Although these methods have demonstrated strong performance and usually outperform conventional signal processing approaches, their effectiveness heavily depends on manually engineered features used during training, a process that is time‐consuming and constrained by researcher‐driven subjectivity, thereby limiting their generalizability across different scenarios (Ruano‐Ordás 2024).

In recent years, deep learning methods have led to major advances in bioacoustics, particularly in the detection and classification of marine species vocalizations. Convolutional neural networks (CNNs) have proven especially effective owing to their ability to automatically extract features from input data during training, thereby reducing the need for manual feature engineering and minimizing human intervention (Krizhevsky et al. 2012). However, CNN‐based approaches typically require preprocessing steps that transform raw time‐domain recordings into the frequency domain using techniques such as the Short‐Time Fourier Transform to generate spectrograms. These approaches have been successfully demonstrated in various studies, including those by Bermant et al. (2019), Thomas et al. (2020), and Schall et al. (2024).

Despite these advancements, challenges remain. Most previous studies have focused on detecting or classifying a single signal type at a time, such as classifying only dolphin whistles or only dolphin clicks. Although these approaches have led to significant advances, they do not capture the full complexity of the marine acoustic environments. In reality, the ocean represents a highly complex soundscape, where anthropogenic and natural sources produce overlapping signals. For example, marine species vocalizations may overlap with calls from other species or with various natural sounds, making single‐class classification systems inadequate for comprehensive ecological monitoring.

These limitations have motivated researchers to develop systems capable of simultaneously detecting and classifying multiple signal types. Previous studies have partially addressed this challenge. Specifically, Duan et al. (2022) developed a CNN‐based approach that could identify and classify multiple calls from bottlenose dolphins, dugongs, and humpback dolphins. Similarly, Hamard et al. (2024) applied a Faster R‐CNN with a ResNet50 backbone to simultaneously detect and classify different vocalizations within the same species, such as dolphin clicks, buzzes, and whistles. While effective, these models do not address mixed events, which limits their applicability in more complex acoustic environments. Therefore, advancing methods that can also handle mixed signals is crucial for capturing the complexity of marine soundscapes and enabling effective large‐scale ecosystem monitoring.

In this study, we propose a deep learning framework for detecting and classifying marine species vocalizations that accounts for the true complexity of underwater environments. Specifically, our study builds upon the YOLO (You Only Look Once) architecture, which is renowned for its high speed and efficiency in object detection and classification tasks. We adapt the architecture to bioacoustic data by representing vocalization events as objects in two‐dimensional spectrogram images, enabling the simultaneous localization and classification of multiple, potentially overlapping signal events.

Alternative detection and classification approaches, including sequence‐based models and transformer‐based architectures, have previously demonstrated strong performance by modeling temporal dynamics in acoustic signals (Hagiwara 2022; Schäfer‐Zimmermann et al. 2024). While powerful, these models often require higher computational resources and longer training times, which can limit their applicability in large‐scale, continuous monitoring scenarios. In contrast, spectrogram image‐based detectors, such as YOLO, offer a favorable balance between detection speed and localization accuracy. Although such approaches may sacrifice some localization precision compared to alternative paradigms, YOLO's computational efficiency enables rapid detection of multiple vocalization events, which can be advantageous in acoustic monitoring environments where real‐time responsiveness is important.

However, a major challenge in developing YOLO‐based frameworks is the limited availability of suitable training data. Ideally, continuous monitoring recordings that capture interactions among various marine species would provide the most realistic foundation for model development. However, this study relies on open‐source recordings, which primarily contain isolated, single‐class vocalizations rather than multi‐species recordings. To address this limitation, we construct synthetic training datasets by combining single‐species recordings to generate mixtures with controlled levels of temporal overlap. These synthetic soundscapes approximate real monitoring conditions and allow us to systematically evaluate the proposed framework under varying degrees of signal overlap.

## 2 Dataset Acquisition and Preparation

### 2.1 Watkins Marine Mammal Sound Database

The dataset used in this study consists of acoustic recordings from various marine species obtained from the Watkins Marine Mammal Sound Database, developed by the Woods Hole Oceanographic Institution (WHOI). The Watkins Marine Mammal Sound Database is one of the most widely used open‐source resources in marine bioacoustics research, as it contains one of the largest publicly available collections of annotated marine species vocalizations, compiled over several decades of fieldwork (Sayigh et al. 2016). The repository contains over 13,000 recordings from more than 60 marine mammal species, each accompanied by detailed metadata such as species name, recording date, geographic location, and vocalization description, enabling species comparisons and serving as a valuable reference for vocal characterization.

For this study, only a subset of species was selected from the database. While the database contains recordings from a wide range of marine species, this study aims to establish a novel deep learning‐based framework, and the focus was therefore placed on defining a reliable baseline rather than addressing unnecessary data complexity at this stage. Therefore, to maintain a focused and manageable dataset, four representative species were selected: Bowhead Whale, Humpback Whale, Walrus, and Sperm Whale. These species were selected for their distinct vocal behaviors, which include the low‐frequency calls of bowhead whales (Cummings and Holliday 1987), the impulsive clicks of sperm whales (Goold and Jones 1995), the complex songs of humpback whales (Au et al. 2006), and the pulsed and knocking sounds of walruses (Larsen and Reichmuth 2021).

**FIGURE 1:**
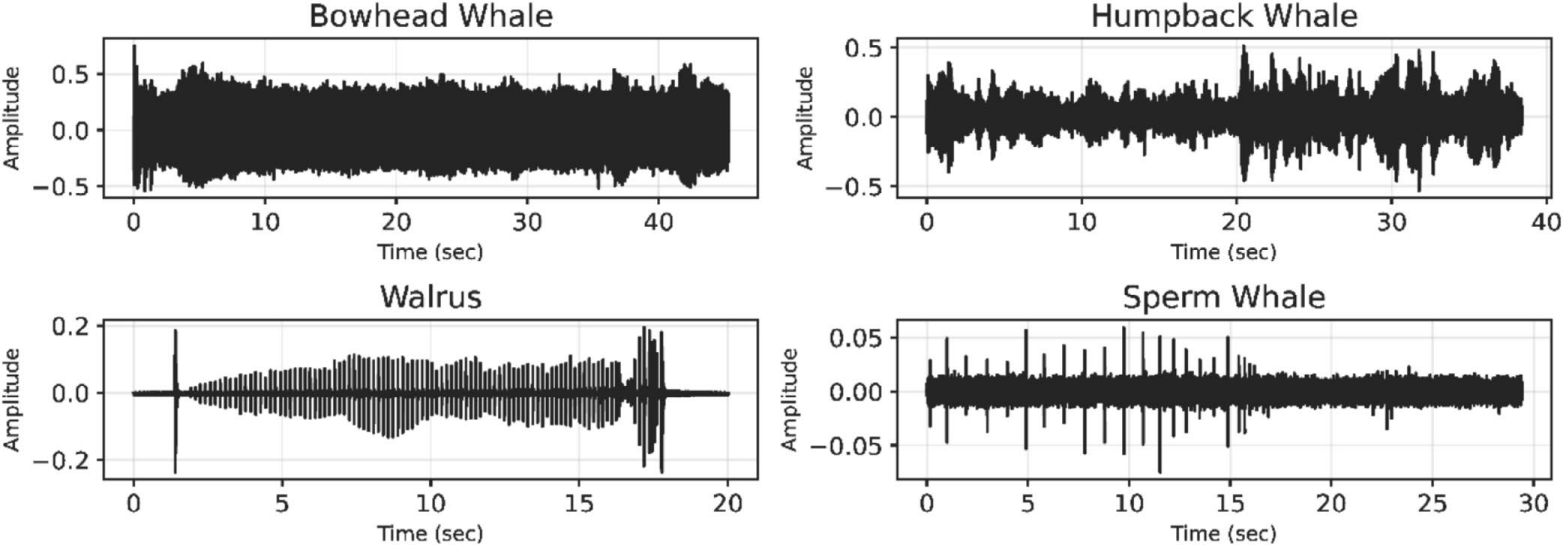
Sample raw waveforms of the four target species (Bowhead Whale, Humpback Whale, Walrus, and Sperm Whale), obtained from the Watkins Marine Mammal Sound Database. The *x*‐axis represents time (seconds), and the *y*‐axis represents signal amplitude.

Figure 1 presents sample recordings from the four species selected for this study and illustrates the type of data available in the repository. The raw recordings were provided in standard WAV format, allowing compatibility with most digital signal processing tools. The audio files were imported into Python using the Librosa library, a widely used framework for audio signal analysis (McFee et al. 2015). Librosa enables efficient reading and manipulation of waveform data, including tasks such as resampling, amplitude normalization, and time–frequency conversion, making it a popular choice for bioacoustic signal analysis.

### 2.2 Data Preprocessing

Raw underwater acoustic recordings usually contain substantial variability in sampling characteristics and signal amplitude, which makes them unsuitable for direct use in deep learning models. Therefore, preprocessing steps were applied to the raw recordings to construct a consistent and reliable dataset for training and evaluation. These steps included segmentation of long recordings, standardization of sampling rates, and amplitude normalization. Each of these steps helped reduce unwanted variability while preserving the essential acoustic characteristics of marine species vocalizations.

#### 2.2.1 Segmentation of Raw Recordings

Table 1 provides an overview of the dataset used in this study, including the number of raw recordings, total recording duration, and original sampling frequencies of the four target species. First, the limited availability of raw recordings poses a challenge, as a small training dataset can hinder model performance. To address this limitation, the raw recordings, particularly those of longer duration, were manually segmented to increase the number of data samples.

**TABLE 1:** Overview of the dataset used in this study, including the number of raw recordings, total recording duration, and original sampling frequencies for the four target species.

Species	Number of raw recordings	Total duration (s)	Original sampling frequency (kHz)
Bowhead whale	57	3815.56	Varies by dataset, ranging from approximately 5 to 82 kHz
Humpback whale	64	5821.74	
Walrus	38	134.24	
Sperm whale	75	849.56	

Segmentation was performed through manual auditory inspection of the recordings, during which recordings were fully reviewed, and segments were extracted whenever a clearly distinguishable vocalization event could be identified. Segment durations were set to a minimum length of 1 s to ensure meaningful frequency representation, as shorter segments may not sufficiently capture the spectral characteristics of the vocalization. Additionally, regions consisting solely of background noise or acoustically ambiguous signals were excluded. This procedure was applied consistently across all recordings, ensuring that only meaningful audio data was used for training. Table 2 summarizes the total number of segmented signals obtained after preprocessing.

**TABLE 2:** Total number of signal segments for the four target species after the segmentation process.

Species	Number of signal segments
Bowhead whale	640
Humpback whale	1625
Walrus	543
Sperm whale	1371

#### 2.2.2 Signal Resampling and Amplitude Normalization

Additionally, the original recordings were sampled at varying frequencies, which could introduce inconsistencies in spectral resolution across the dataset during training (Mellinger 2004). To address this, all recordings are usually resampled to a uniform frequency. Determining the optimal sampling frequency is challenging, as lower rates may fail to capture important acoustic details, while higher rates increase computational cost. In our study, after several experimental evaluations, we selected a standardized sampling frequency of 40 kHz as a compromise between preserving information and maintaining computational efficiency.

**FIGURE 2:**
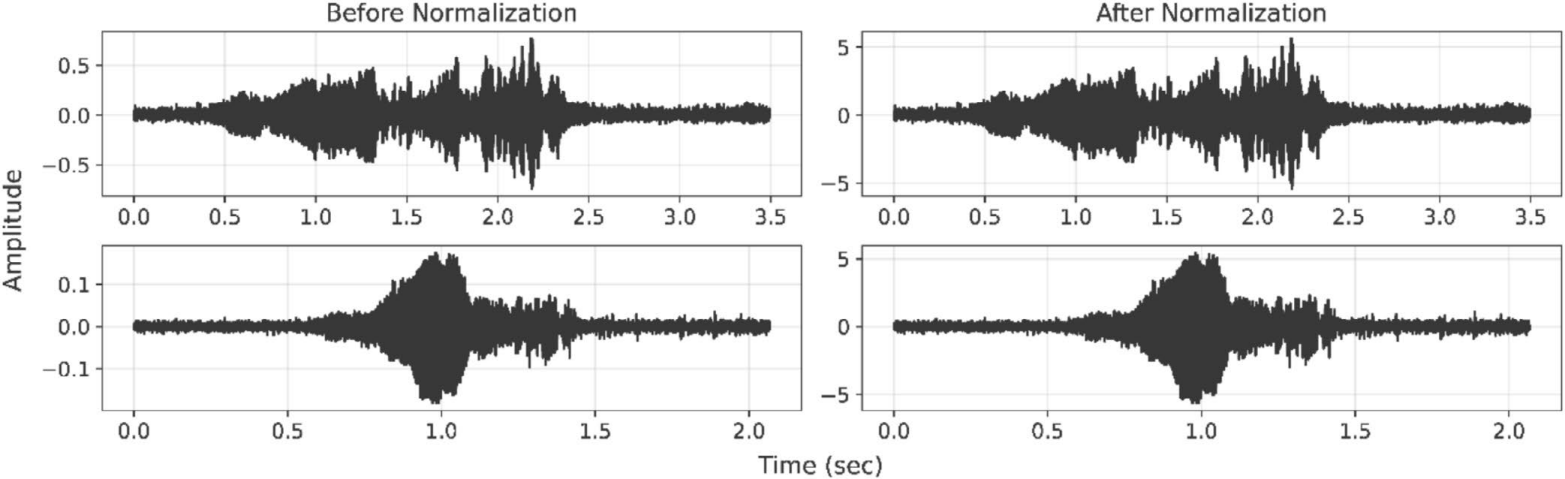
Two sample signals from the dataset before (left) and after (right) root mean square (RMS) normalization.

Following resampling, since the dataset consists of signals recorded at various locations, a data normalization process was applied to address amplitude variability arising from differences in acquisition processes and recording equipment (Singh and Singh 2020). In this study, root mean square (RMS) normalization was applied to rescale each waveform, ensuring that all recordings had comparable amplitude ranges. This allows the model to focus on relevant acoustic patterns while minimizing the influence of varying recording conditions. Figure 2 presents sample signals from the dataset, illustrating their waveforms before and after RMS normalization.

### 2.3 Synthetic Monitoring Dataset Construction

The recordings used in this study consist of single‐class vocalizations, with each signal containing the call of only one species. To simulate realistic monitoring conditions required for model training, these isolated signals were combined to create synthetic monitoring datasets. Two types of datasets were generated: (Amorim et al. 2022) non‐overlapping datasets, in which vocalizations from different species are randomly arranged sequentially within a predefined time window, and (Au et al. 2006) overlapping datasets, in which two or more signals are superimposed to mimic simultaneous vocalizations from multiple species.

#### 2.3.1 Non‐Overlapping Monitoring Dataset

To establish a baseline for model performance, we first created a non‐overlapping monitoring dataset for training and validation. The generation process begins by selecting a random subset of unique classes from the available data, ensuring that each synthetic monitoring sample includes a diverse combination of species. From each selected class, one signal is then randomly chosen and prepared for inclusion in the mixture.

The selected signals are then randomly placed within a fixed time window of 5 s. This duration was chosen because most segmented recordings were approximately 1 s long, allowing multiple events to be arranged within a single monitoring segment without significantly reducing time resolution. Additionally, small random gaps are added between signals to ensure that no signals overlap in time and to mimic the natural pauses that often occur between animal vocalizations in underwater recordings.

Once all signals are positioned, they are combined to form a single continuous waveform representing a synthetic monitoring sample. For each sample, the species type, class label, and the start and end times of every signal are recorded. This information serves as the primary reference for each mixture and is later used to generate the annotation files required for training and validating the proposed model.

Finally, Gaussian noise is added uniformly across the entire waveform to simulate the ambient acoustic conditions of underwater environments. Because each signal originally contains background noise specific to its recording location and conditions, adding additional noise helps mask these variations and creates a more homogeneous acoustic environment. This step ensures that all signals within a mixture appear as if they were captured simultaneously in the same recording setting.

**FIGURE 3:**
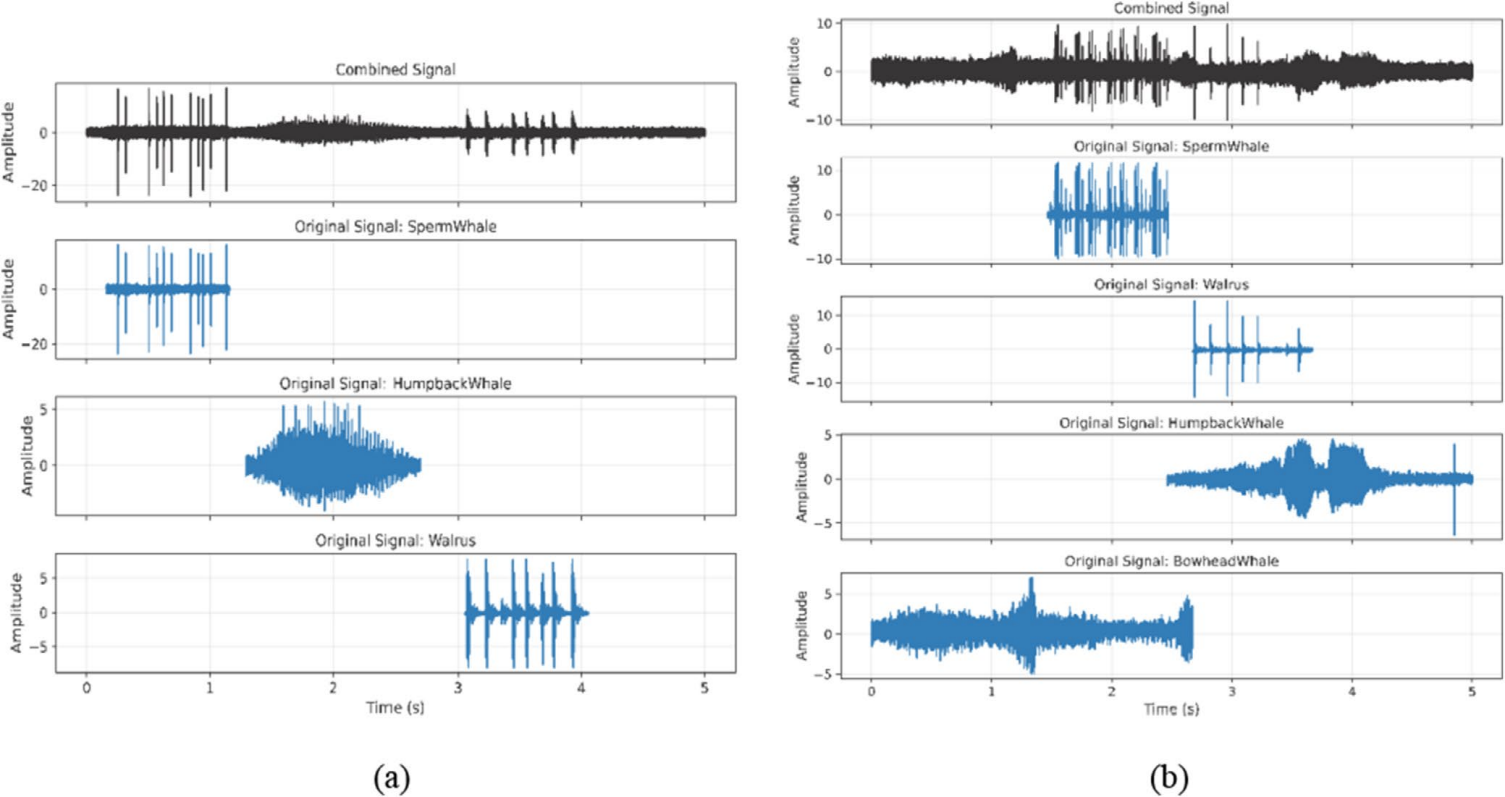
Example of a synthesized (a) non‐overlapping monitoring sample and (b) overlapping monitoring sample containing multiple species vocalizations. The black waveform represents the final combined mixture, while the blue waveforms indicate the individual species signals used to construct the mixture, illustrating their temporal placement within the five‐second monitoring window.

Gaussian noise is initially generated using a zero mean and unit variance distribution across the 5 s mixture duration. The RMS of the clean waveform, consisting of the combined vocalization signals prior to noise addition, is then computed. Given a predefined signal‐to‐noise ratio (SNR), the target noise RMS is calculated from the clean waveform RMS according to:

(1) $$20\log_{10}\left(\frac{{\mathrm{RMS}}_{\text{clean}}}{{\mathrm{RMS}}_{\text{noise}}}\right)={\mathrm{SNR}}_{\text{target}}$$

where RMS_clean_ is the RMS of the clean waveform, RMS_noise_ is the target Gaussian noise RMS, and SNR_target_ is the predefined SNR. In this study, SNR_target_ was set to 4 dB. This setting reduces the influence of the inherent background noise while preserving the detectability of vocalization events. The initially generated Gaussian noise is then rescaled to match the target RMS and added uniformly across the waveform. Finally, an additional RMS normalization step is applied to the resulting noisy waveform to ensure consistent amplitude levels across all monitoring samples. Through these processes, synthetic monitoring samples were generated, containing multiple non‐overlapping vocalizations embedded within a consistent background noise level. This approach results in a well‐structured and acoustically coherent dataset suitable for baseline model training and validation. Figure 3a illustrates an example of a synthesized non‐overlapping monitoring sample.

#### 2.3.2 Overlapping Monitoring Dataset

To evaluate the model under more challenging acoustic conditions, an overlapping monitoring dataset was also created to simulate the true complexity of underwater soundscapes. The overall procedure is similar to that of the non‐overlapping case, with the key difference being that events are randomly combined to overlap in time within the selected time window (5 s). However, to prevent excessive stacking of signals in the same region, an inhibition mechanism is implemented to limit the number of signals allowed to overlap within the same region. Specifically, a constraint is enforced such that no more than two signals may overlap at any given time. While an individual signal may overlap with multiple other signals at different time intervals, the number of simultaneously overlapping signals at any time point is strictly limited to two. Figure 3b illustrates an example of a synthesized overlapping monitoring sample containing multiple vocalizations that overlap in time and are embedded within a consistent background noise level.

## 3 Methodology

### 3.1 Short‐Time Fourier Transform

The Fourier Transform is one of the most fundamental tools in signal analysis, revealing spectral information that is usually difficult to analyze from raw waveform data alone (Brigham and Morrow 1967). It works by decomposing signals into their constituent sinusoidal components, showing how energy is distributed across different frequencies. Given a discrete, finite signal *x*[*n*], the Fourier Transform is defined as the following:

(2) $$X\left[k\right]=\sum_{n=0}^{N-1}x\left[n\right]e^{-j\frac{2\pi }{N}\mathrm{kn}},\;k=0,1,\ldots ,N-1$$

This formulation helps create a connection between time and frequency domains, making the Fourier Transform essential for analyzing signal structure and spectral characteristics across various applications, including bioacoustics.

However, the Fourier Transform has a significant limitation in that it provides the overall frequency information of a signal, but not when those frequencies occur. This limitation is very critical, especially for marine species vocalization, in which signals are nonstationary and vary very rapidly in time. To address this, the Short Time Fourier Transform (STFT) was developed, which applies a sliding window across the signal to preserve both frequency and time information, making it one of the most widely used time‐frequency analysis techniques, especially in bioacoustics (Qian and Chen 1999).

The STFT works by dividing the signal into overlapping segments of fixed length using a window function. The Fourier Transform is then computed for each individual segment, revealing the frequency content present during that specific time interval. Given a discrete, finite signal *x*[*n*], the STFT is defined as the following:

(3) $$X\left[k,m\right]=\sum_{n=0}^{N-1}x\left[n+\mathrm{mR}\right]w\left[n\right]e^{-j\frac{2\pi }{N}\mathrm{kn}}$$

where *m* is the time frame index, *k* is the frequency bin index, and *R* is the hop size parameter (the number of samples by which the window advances between successive frames).

The resulting STFT can be visualized as a spectrogram, a two‐dimensional representation of the signal. In this visualization, the horizontal axis represents time, the vertical axis represents frequency, and the intensity or color indicates the magnitude of each frequency component, making temporal and spectral patterns clearly visible (Khodzhaev 2024). In practice, the Python Librosa library provides built‐in STFT and spectrogram plotting functions, allowing one to visualize spectrograms much more easily without the need to manually implement the STFT formula as shown in Equation (3).

**FIGURE 4:**
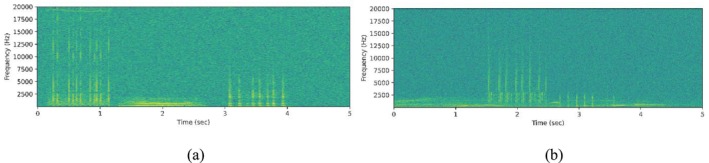
STFT‐based spectrogram representations of (a) the non‐overlapping monitoring sample and (b) the overlapping monitoring sample shown in Figure 3a,b, respectively. Spectrograms were generated using a window length of 1024 samples, a hop length of 256 samples, and a Hann window function, and were converted to dB scale for visualization.

In this study, STFT‐based spectrograms were used as input representations for model training and evaluation. Specifically, the synthetic monitoring samples were converted into spectrograms using STFT to capture both the temporal and spectral characteristics of marine species vocalizations. To ensure consistency across all samples, the same STFT parameters were used throughout the study, including a window length of 1024 samples, a hop size of 256 samples, and a Hann window function. The resulting spectrogram magnitudes were converted to a dB scale and represented on a linear‐frequency axis. Figure 4 illustrates the corresponding spectrogram representations of the non‐overlapping and overlapping monitoring samples shown in Figure 3, generated using the predefined STFT parameters.

### 3.2 Proposed YOLO‐Inspired Deep Learning Framework

The proposed framework in this study is inspired by the YOLO (You Only Look Once) architecture. YOLO is a single‐stage object detection framework optimized for real‐time image analysis (Redmon et al. 2016). Unlike traditional two‐stage approaches that separate region proposal and classification, YOLO formulates detection as a unified regression problem, simultaneously predicting object locations and class probabilities in a single forward pass. This is achieved by dividing the input image into a grid, where each grid cell learns spatially localized predictions, enabling high computational efficiency while maintaining strong detection performance. Owing to its end‐to‐end design and fast inference speed, YOLO has been widely adopted in applications that require scalable, real‐time detection.

**FIGURE 5:**
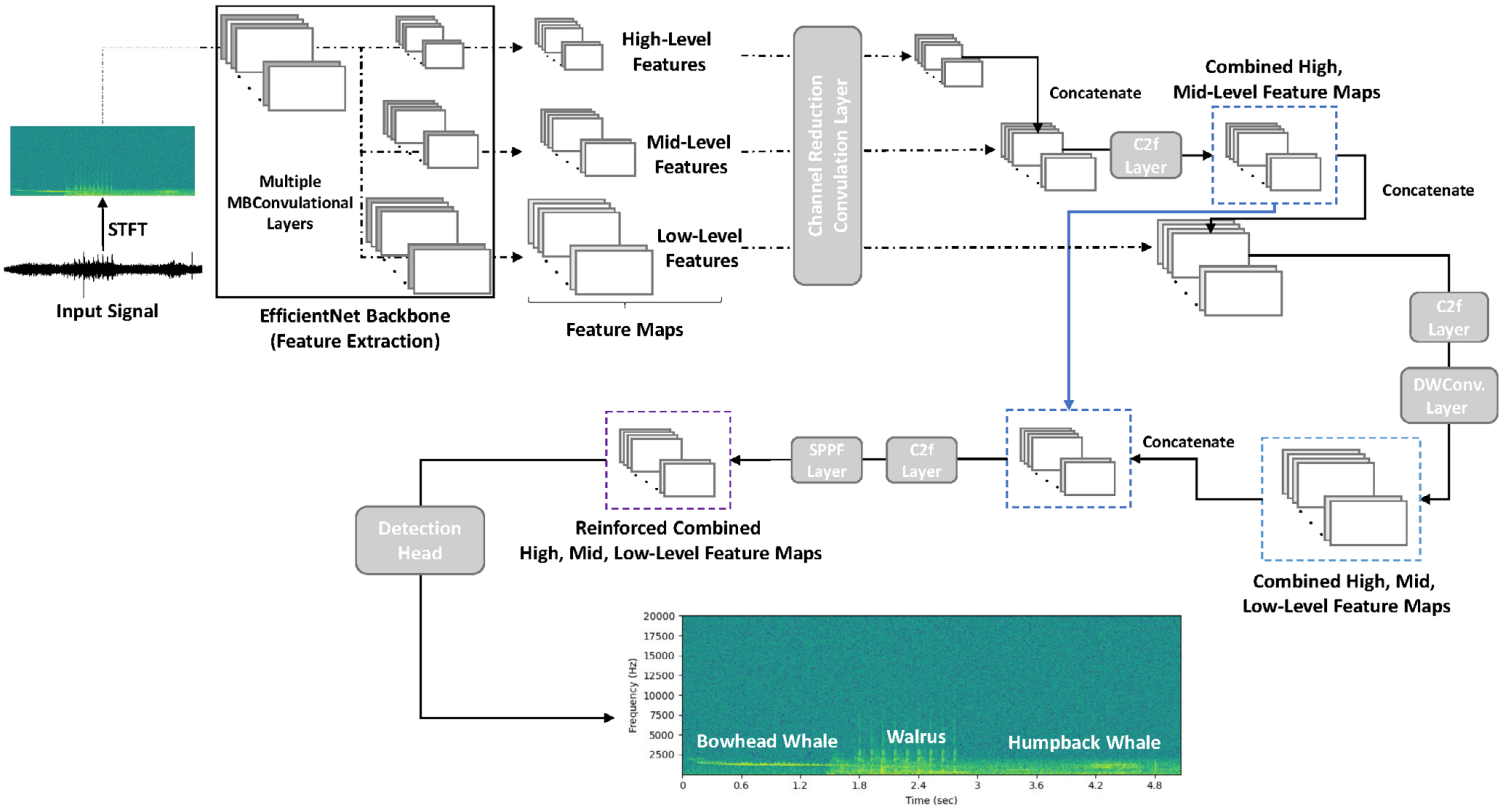
Overview of the proposed YOLO‐inspired deep learning framework for marine species vocalization detection. The model processes STFT‐based spectrogram inputs through an EfficientNet‐B3 backbone for multi‐scale feature extraction, followed by multi‐scale feature fusion using FPN and PAN structures with C2f modules. The fused feature maps are then passed to detection heads that jointly predict localization and species classification.

Building on the efficient detection framework of YOLO, this study adapts the architecture for spectrogram‐based acoustic event detection, where the objective is to identify the presence and timing of marine species vocalizations. Figure 5 illustrates the overall structure of the proposed YOLO‐inspired deep learning‐based framework. The model processes STFT‐based spectrogram images as two‐dimensional inputs and follows a unified detection pipeline consisting of three main stages: multi‐scale feature extraction, feature fusion, and final event prediction.

First, feature extraction is performed to capture the acoustic characteristics of each vocalization event within the spectrogram, which are used to predict its timing and species identity. This is performed using a pretrained EfficientNet‐B3 backbone, which serves as the primary feature extractor for the spectrogram inputs. EfficientNet is a family of CNN architectures designed to balance model accuracy and computational efficiency, outperforming earlier architectures such as ResNet or MobileNet at comparable computational cost (Tan and Le 2019). Originally trained on large‐scale image datasets, the EfficientNet backbone is capable of capturing both low‐level and high‐level feature patterns (Baowaly et al. 2024). Feature maps are extracted at three different scales (low, mid, and high level), allowing the model to preserve fine details as well as broader contextual patterns.

Following feature extraction, the model integrates the extracted multi‐scale features through a combination of top–down and bottom–up fusion paths inspired by the Feature Pyramid Network (FPN) and Path Aggregation Network (PAN) architectures, respectively (Lin et al. 2017; Liu et al. 2018). In this process, C2f modules (Cross Stage Partial with dual feature fusion) serve as the primary component for feature fusion. The C2f block divides the input feature map into two branches: one undergoes a series of convolutional transformations, while the other bypasses this processing. The two branches are then concatenated and fused through a final convolution, promoting efficient gradient propagation and partial feature reuse. The use of C2f modules offers several key advantages over conventional convolutional structures by enhancing network stability during training, strengthening local feature extraction, and improving memory efficiency, thereby enabling the model to handle large inputs effectively with minimal loss in detection accuracy (Li et al. 2024).

**FIGURE 6:**
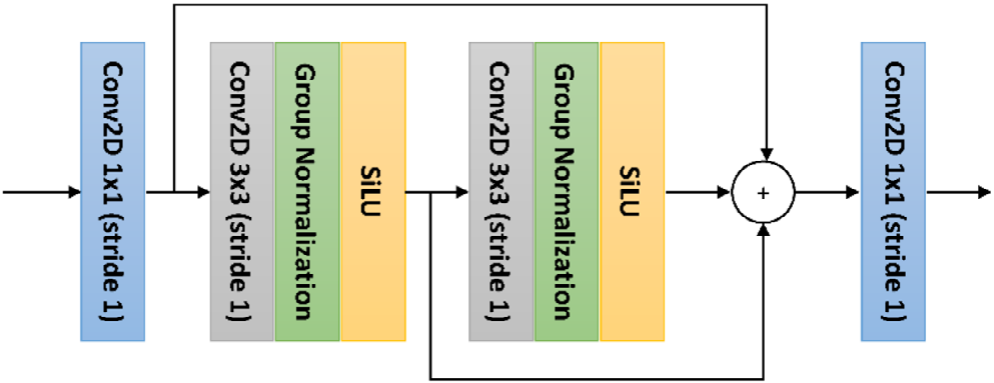
Structure of the C2f (Cross Stage Partial with dual feature fusion) module used in the proposed model. The input feature map is split into two branches, where one branch undergoes a series of convolutional transformations while the other bypasses this processing. The resulting feature maps are then concatenated and fused through a final convolution layer.

Figure 6 illustrates the C2f module used in the proposed model. In the top–down FPN pathway, C2f modules combine broad features from the low‐resolution layers with fine details from the high‐resolution maps, improving the model's ability to localize short acoustic events in time. In the bottom‐up PAN pathway, additional C2f modules pass high‐resolution information upward, helping to preserve subtle spectral information important for identifying marine species calls. Together, this dual‐path fusion enables the architecture to balance spectral and temporal precision, leading to a more improved detection performance across different time scales.

Finally, the fused feature maps are passed to the detection heads, which separately handle signal detection, classification, and confidence estimation. However, in contrast to conventional two‐dimensional detection heads that predict spatial bounding boxes for image data, our approach adapts the detection heads for one‐dimensional temporal event detection. Here, one‐dimensional detection refers to predicting only the temporal boundaries (start and end times) of each event along the time axis of the spectrogram, without estimating its frequency‐band extent.

This design choice reflects the nature of spectrogram‐based analysis, in which approximate frequency ranges for many marine species' vocalizations are often known in advance from prior studies and can also be refined during the annotation process, as was done in this study. While absolute frequencies may vary due to individual differences, these characteristic frequency ranges tend to remain relatively consistent, and small frequency shifts do not alter the semantic identity of a call. Accordingly, the proposed framework does not attempt to predict frequency bands during detection. Instead, it focuses on temporal localization, while frequency content is implicitly learned through spectrogram‐based feature extraction.

### 3.3 Annotation

Unlike conventional classification models, YOLO requires annotations that precisely specify the spatial location of each class signal within the spectrogram image. Traditional approaches typically rely on manual annotation, where experts visually inspect spectrograms and draw bounding boxes around vocalizations of interest. Although manual annotation is the most reliable and often the preferred method, it is highly time‐consuming, labor‐intensive, and prone to human subjectivity, especially when applied to large volumes of marine acoustic recordings. To address these limitations, our study implements an automated annotation pipeline designed to generate YOLO‐compatible annotations directly from the spectrogram representations. Unlike conventional annotation methods that operate on rendered spectrogram images (PNG, JPG files, etc.), the proposed method works on the raw STFT‐based spectrogram matrices, in which annotation boundaries are determined based on acoustic magnitude values.

#### 3.3.1 Annotation for Non‐Overlapping Monitoring Dataset

The basic principle of this automated annotation method is that core vocalizations are characterized by amplitude values that are substantially higher than the surrounding background noise. To exploit this property, for each synthesized monitoring sample, the corresponding spectrogram was first analyzed to estimate the background noise level. In this study, the background noise level was defined using the median magnitude, as the median provides a robust measure that is less affected by outliers originating from high‐energy regions.

Using the known start and end times of each vocalization event, the temporal boundaries (*x*‐axis) of each event were first defined within the spectrogram. Within each temporal window, an amplitude‐based thresholding approach was applied to identify frequency bins exhibiting sustained energy above the estimated background level. In this study, an adaptive criterion was employed in which the threshold was defined as the larger of 3 dB or 2.5 times the median absolute deviation from the estimated background, allowing the method to adapt to variations in background noise and signal characteristics across samples.

**FIGURE 7:**
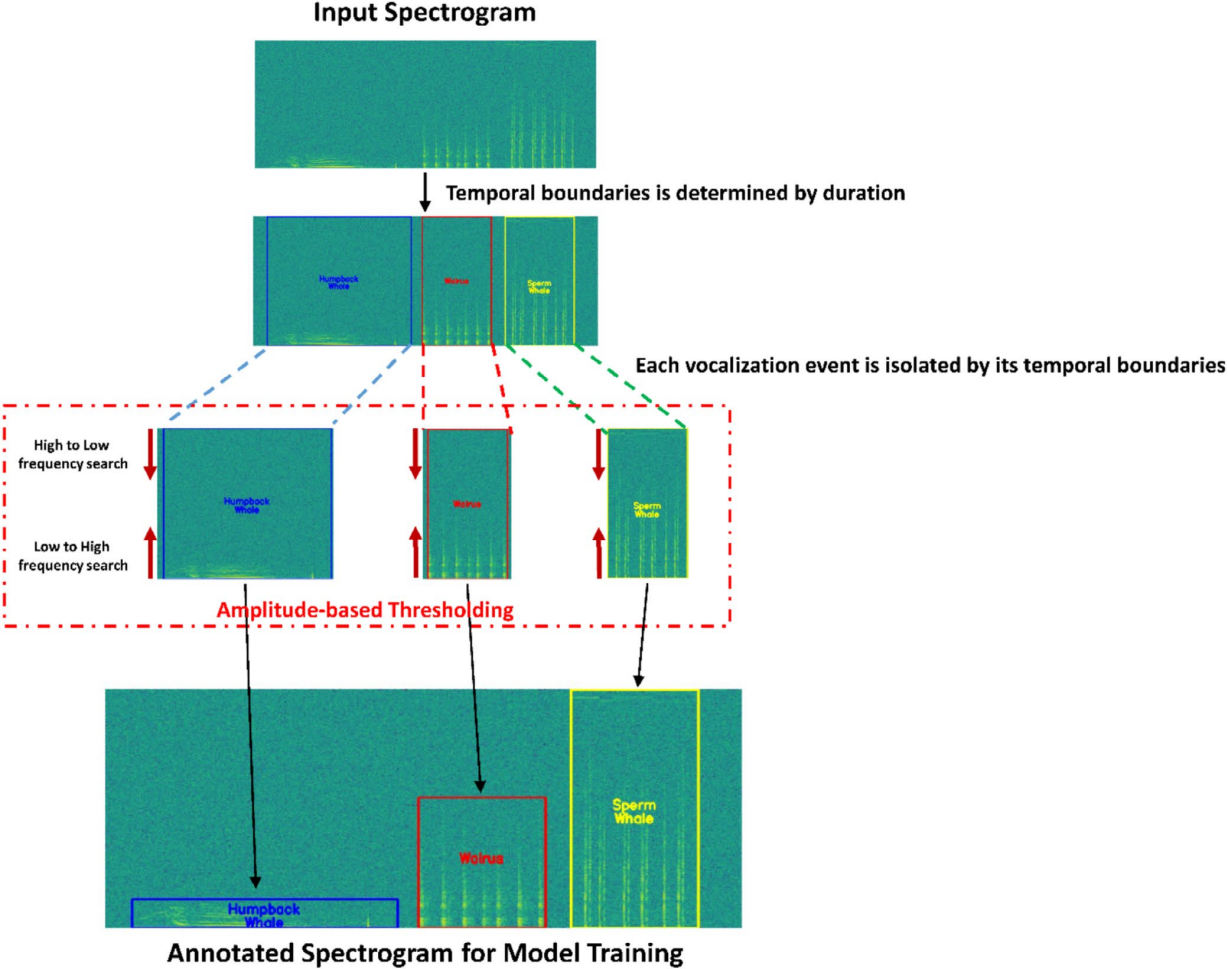
Overview of the automated annotation pipeline for the non‐overlapping monitoring dataset. Using the known temporal boundaries of each inserted vocalization event, corresponding spectrogram frames are identified. An amplitude‐based thresholding procedure is then applied to the raw STFT spectrogram matrix to detect frequency bins exhibiting energy above the estimated background level. The resulting high‐energy region defines the upper and lower frequency bounds, which are combined with the temporal boundaries to generate YOLO‐compatible bounding box annotations.

To determine the frequency extent of each event, a bidirectional search was performed along the frequency axis. Specifically, one search proceeded from high to low frequencies and another from low to high frequencies. For each frequency bin, the average magnitude across the temporal window was computed, and the search was terminated when this value first exceeded the predefined threshold. Figure 7 illustrates the proposed automated annotation process implemented in this study for the non‐overlapping monitoring dataset. To ensure the reliability of the automated annotation process, all generated annotations were visually reviewed, and it was confirmed that the detected temporal and frequency boundaries aligned with the expected vocalization regions.

#### 3.3.2 Annotation for Overlapping Monitoring Dataset

**FIGURE 8:**
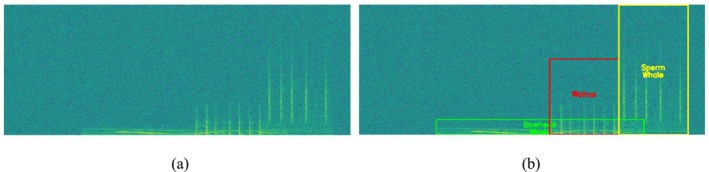
Automated annotation procedure for the overlapping monitoring dataset. (a) STFT‐based spectrogram of a synthesized overlapping monitoring sample containing multiple simultaneous vocalizations. (b) Corresponding annotation results, where temporal boundaries are determined from known insertion times and frequency extents are defined using the mean bounding box height calculated for each species from the non‐overlapping dataset.

Unfortunately, the proposed automated annotation method is applicable only to cases in which signals do not overlap in time, as overlapping signals produce mixed energy regions, in which distinguishing individual calls based solely on pixel intensity contrast is much more difficult. Thus, the frequency boundaries for overlapping vocalizations are determined using an average boundary height. Specifically, the mean bounding box height for each species is calculated from the non‐overlapping instances and used as the representative vertical boundary for overlapping segments. This assumption is based on the observation that, within the same species, vocalizations of a similar call type generally occupy a consistent frequency range. Figure 8 illustrates the annotation process implemented in this study for the overlapping monitoring dataset.

However, it is important to note that even within a single species, different call types may vary significantly in their frequency distribution. For example, low‐frequency tonal calls and high‐frequency echolocation clicks may occur within the same species but occupy distinct spectral bands (Zahn et al. 2021). In the present study, the dataset primarily consisted of recordings containing a single dominant call type per species. Under this constraint, the use of average frequency boundary heights remained appropriate and effective.

### 3.4 CutMix

To improve model performance, CutMix, a data augmentation technique commonly used in image‐based deep learning frameworks, was implemented in this study to enhance dataset diversity. Originally introduced by Yun et al. (2019), CutMix is an augmentation strategy in which a rectangular region is randomly cut from one image and pasted onto another, producing new training samples that contain partial content from multiple sources, increasing variation in object appearance and spatial configuration. By exposing the model to images in which objects may be partially hidden or appear in different positions, CutMix encourages the network to focus on informative local features rather than relying on the general image as a whole, improving classification performance.

In our study, a modified version of CutMix was developed and adapted for spectrogram‐based acoustic data. The proposed CutMix implementation operates at the object (signal) level, where the cut region is determined by the previously annotated bounding boxes and not randomly selected. Specifically, the CutMix process begins by randomly selecting a donor spectrogram and a base spectrogram from the training set. From the donor spectrogram, one signal event is randomly selected from the annotated vocalizations present. The selected signal is then extracted using its corresponding bounding box coordinates and pasted onto the base spectrogram.

**FIGURE 9:**
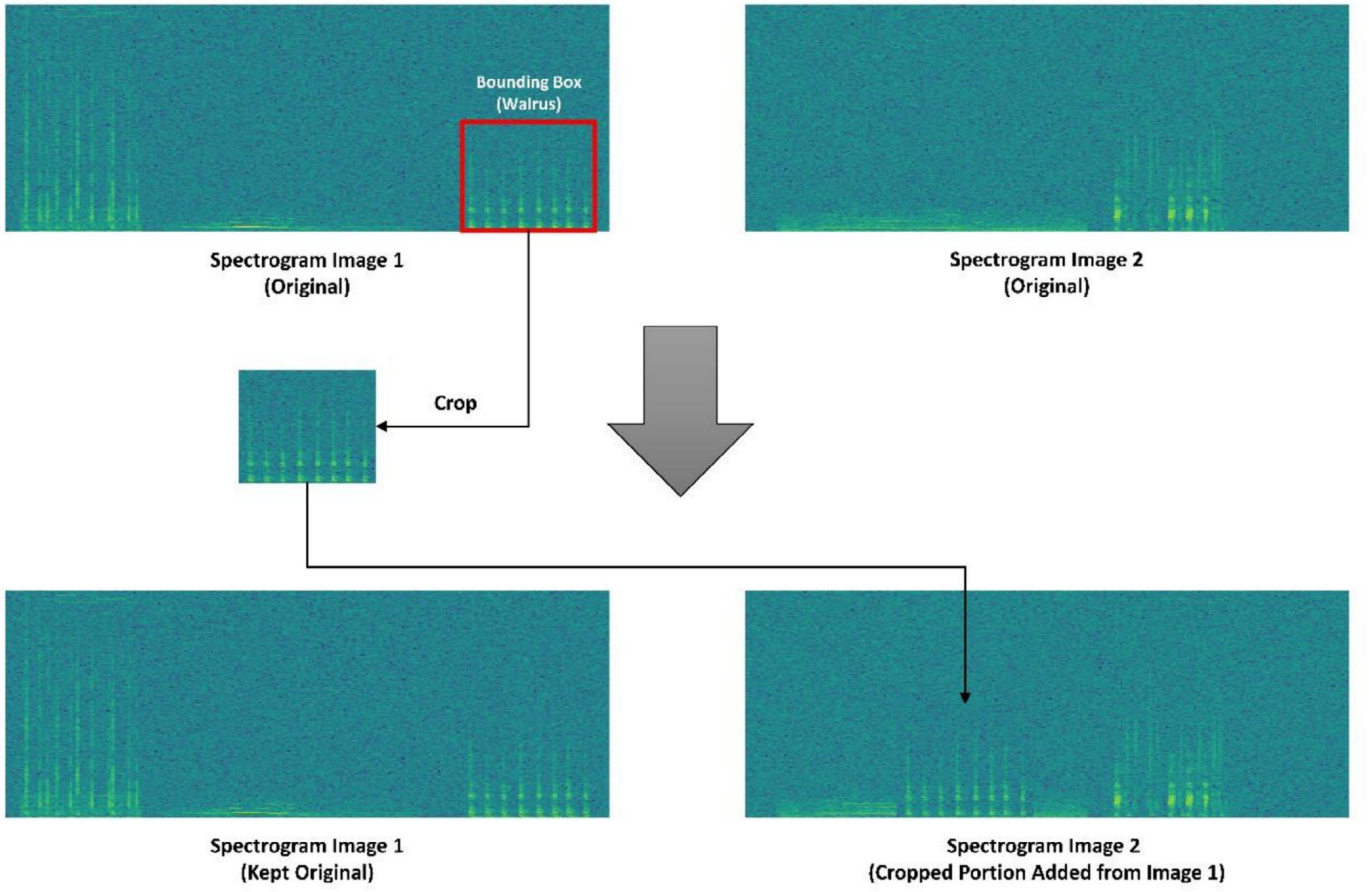
Overview of the modified CutMix procedure implemented in this study. Annotated vocalization regions are cropped from a donor spectrogram based on existing bounding boxes and pasted into a base spectrogram using random horizontal shifts along the time axis.

However, unlike the original CutMix formulation, which allows cropped regions to be placed at arbitrary spatial locations, the proposed method restricts placement to random horizontal shifts along the time axis only, and vertical shifts along the frequency axis were intentionally avoided. Although the detection framework focuses on one‐dimensional temporal localization, including random vertical shifts would introduce unrealistic spectral distortions, such as placing vocalizations in frequency ranges that are inconsistent with known biological characteristics of the species. Such distortions could negatively affect training stability and lead to misleading validation outcomes, as frequency information is still implicitly learned during feature extraction. By constraining augmentation in this manner, the generated augmented samples remain acoustically plausible. Figure 9 provides an overview of the modified CutMix method implemented in this study.

## 4 Application

### 4.1 Dataset Split for Training and Testing

The proposed methodology was applied to both the non‐overlapping and overlapping monitoring datasets to evaluate model performance. When developing machine learning or deep learning models, it is essential not only to achieve high predictive accuracy but also to ensure that the models generalize effectively to new, unseen data. Typically, this process involves dividing the dataset into distinct training and test subsets, where the model is trained and optimized on the training set, and its performance is evaluated on the separate, unseen test set.

In the previous section, we introduced the process of constructing the synthetic monitoring dataset for both non‐overlapping and overlapping, describing how individual signals were combined to simulate realistic acoustic monitoring environments. However, before generating this dataset, the single‐signal dataset (Table 2) was first divided into independent training and test subsets to ensure that the trained deep learning model remains unbiased during evaluation.

In this study, the single‐signal dataset train‐test split was conducted using a stratified split with a 60:40 ratio to ensure that both subsets contained all classes in proportions representative of the original data (Pedregosa et al. 2011). Although this split ratio is less common than conventional ratios such as 70:30 or 80:20, we adopted a larger test set due to the limited number of raw recordings available per species. This allowed for a more reliable and robust evaluation of model performance across classes.

However, since the dataset consists of signals segmented from raw recordings, segments originating from the same recording may contain vocalizations produced by either the same individual or multiple individuals. If segments from the same individual are divided between the training and test sets, this can result in data leakage, leading the model to overestimate its performance due to similarities shared across the two sets. To minimize this risk, the stratified split was conducted such that all segments derived from the same raw recording were exclusively assigned to either the training or the test set.

The synthetic monitoring datasets were then generated separately from the two single‐signal dataset partitions to prevent any potential data leakage between the training and evaluation stages. Specifically, the training monitoring dataset was constructed exclusively from signals in the training subset, whereas the test monitoring dataset was constructed exclusively from signals in the test subset. This ensures that no data used during training was present in the test set, thereby guaranteeing that the model's performance metrics accurately reflect its ability to generalize to unseen recordings.

### 4.2 Model Training

During training, the model was trained using an ensemble strategy based on multiple complementary partitions of the training data. The training set was partitioned into five folds, and five separate models were trained, each using four folds for training and one fold for validation. This ensured that each training sample was used for validation in one model and for training in the remaining models. The resulting five models were then combined into an ensemble, and their aggregated predictions were used for evaluation on the held‐out test set. Specifically, each model generates a prediction map for a given input sample. The prediction maps are aggregated by elementwise averaging to form a single ensemble prediction map, which is then decoded to generate the final detection results on the unseen test set. This ensemble strategy reduces sensitivity to stochastic factors such as random initialization and fold‐specific data variations, leading to more stable and robust performance estimates.

Model training was carried out using a fixed set of hyperparameters across all folds to ensure consistency and reproducibility. The Efficient‐B3 backbone was initialized with pretrained weights, which were kept frozen throughout the training process. A batch size of 16 was used for both training and validation. Each model was trained for a maximum of 300 epochs with early stopping, where training was halted if no improvement in validation performance was observed for 10 consecutive epochs. Optimization was performed using the AdamW optimizer with a constant learning rate of 0.0005, and a weight decay of 5 × 10^−4^. All experiments were implemented in PyTorch and executed on a single NVIDIA RTX 3090 GPU. Identical hyperparameters and optimization settings were maintained across folds to ensure fair comparison and reduce training‐induced variability.

### 4.3 Model Testing

The trained models with optimal parameters were then evaluated on the unseen test set that had been separated during the initial train–test split stage. For performance assessment, precision, recall, and F‐score metrics were used, as the high class imbalance within the dataset could cause the accuracy metric to overestimate model performance (Sokolova and Lapalme 2009). Unlike accuracy, these metrics provide a more detailed evaluation of the model's ability to correctly identify each class.

Precision measures the percentage of correct positive predictions out of all positive predictions made by the model. Recall measures the percentage of actual positive cases that the model correctly identifies. The F‐score combines precision and recall into a single metric by taking their harmonic mean, providing a balanced measure that accounts for both metrics. The equations for each metric are as follows:

(4) $$\text{Precision}=\frac{\mathrm{TP}}{\mathrm{TP}+\mathrm{FP}}$$

(5) $$\text{Recall}=\frac{\mathrm{TP}}{\mathrm{TP}+\mathrm{FN}}$$

(6) $$F_{\text{score}}=2\times \frac{\text{Precision}\times \text{Recall}}{\text{Precision}+\text{Recall}}$$

where TP denotes true positives, FP false positives, and FN false negatives. A true positive corresponds to a correctly detected and classified vocalization event of a given species, whereas a false positive indicates an incorrectly detected or mislabeled signal, and a false negative represents a missed detection.

In this study, a true positive was determined using an Intersection over Union (IoU) threshold. For each vocalization event, the predicted temporal boundaries were matched with the corresponding ground‐truth boundaries, and a detection was counted as a true positive if the IoU exceeded 50%. Predicted events that did not match any ground‐truth event with IoU ≥ 0.5 were counted as false positives, while ground‐truth events with no matching prediction were counted as false negatives.

In addition, non‐maximum suppression (NMS) was applied to remove multiple predictions corresponding to the same vocalization event. Specifically, when multiple predicted events overlapped by more than 50%, only the prediction with the highest confidence score was retained, and the remaining predictions were suppressed. A confidence threshold was also applied prior to evaluation. For the non‐overlapping monitoring dataset, the confidence threshold was set to 0.5. For the overlapping dataset, considering the increased detection difficulty and the presence of simultaneous events, the confidence threshold was lowered to 0.3 to reduce the risk of suppressing valid overlapping vocalizations.

## 5 Results and Discussion

### 5.1 Model Assessment on Non‐Overlapping Dataset

Table 3 presents the performance metrics obtained when the model was trained and evaluated on the non‐overlapping monitoring dataset. Overall, the model demonstrated strong performance across all species. Precision values were high, indicating that the model produced very few misclassifications and assigned class labels reliably when the detections were made. Recall values were also relatively high for all classes, indicating that the model was able to detect the majority of vocalization events present in the monitoring samples.

**TABLE 3:** Performance metrics when model is trained and evaluated on the non‐overlapping monitoring dataset.

Species	Precision	Recall	F‐score
Bowhead whale	1.000	0.883	0.938
Humpback whale	0.997	0.928	0.961
Walrus	1.000	0.927	0.962
Sperm whale	0.990	0.986	0.988

Sperm whale class exhibited the highest recall, suggesting that these signals were least likely to be missed due to their distinctive characteristics. In contrast, Bowhead whale calls showed comparatively lower recall, indicating that some calls were more difficult to detect, potentially because of their relatively lower signal strength. The resulting F‐scores remained consistently high for all species, reflecting a balanced performance between precision and recall. These results confirm the model's capability for accurate detection and classification under simplified monitoring conditions and establish a strong baseline for subsequent evaluations under more complex overlapping acoustic scenarios.

#### 5.1.1 Sensitivity Improvement via Single‐Class Dataset

**FIGURE 10:**
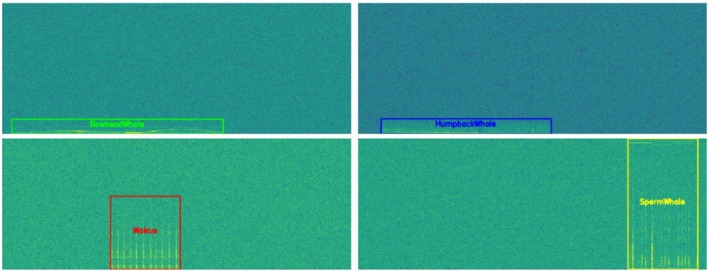
Example of the single‐class monitoring dataset used during training. Each five‐second sample contains one vocalization event from a single species, randomly positioned within the time window.

However, noticing that there was room for improvement, an additional training phase was conducted using a single‐class dataset alongside the non‐overlapping dataset to improve sensitivity. In this configuration, each five‐second monitoring sample contained only one species' vocalization randomly placed within the five‐second duration, allowing the network to focus on learning the representative features of that species without interference from other signals. Figure 10 illustrates the sample single‐class dataset created to be used to train alongside the non‐overlapping dataset.

Table 4 presents the performance obtained after incorporating the single‐class dataset during training. Compared with the baseline results shown in Table 3, the inclusion of the single‐class dataset led to a consistent improvement across all evaluated metrics. Recall increased for all species by an average of approximately 2.8%, indicating an enhanced ability of the model to detect true vocalization events. In particular, the recall of the Bowhead whale showed a substantial improvement of approximately 7.4%, suggesting that the additional single‐class samples helped the model better capture representative features of this species. Precision and F‐score also improved, with average increases of approximately 0.2% and 1.6%, respectively, reflecting more accurate and balanced detection and classification performance.

**TABLE 4:** Performance metrics when model is trained and evaluated on the non‐overlapping monitoring dataset, with the single‐class dataset additionally included during training.

Species	Precision	Recall	F‐score
Bowhead whale	1.000	0.957	0.987
Humpback whale	0.995	0.942	0.968
Walrus	1.000	0.937	0.967
Sperm whale	1.000	0.992	0.996

**FIGURE 11:**
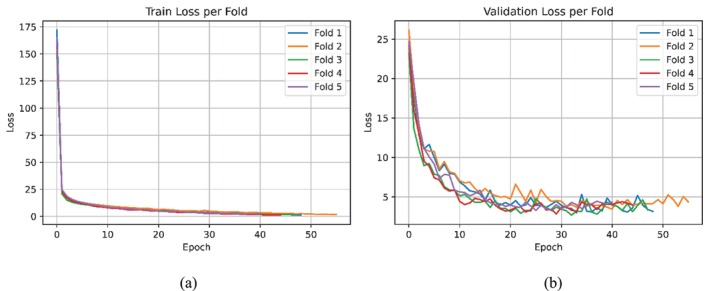
Loss curves for the non‐overlapping monitoring dataset before incorporating the single‐class dataset: (a) Training loss and (b) validation loss across fivefold.

**FIGURE 12:**
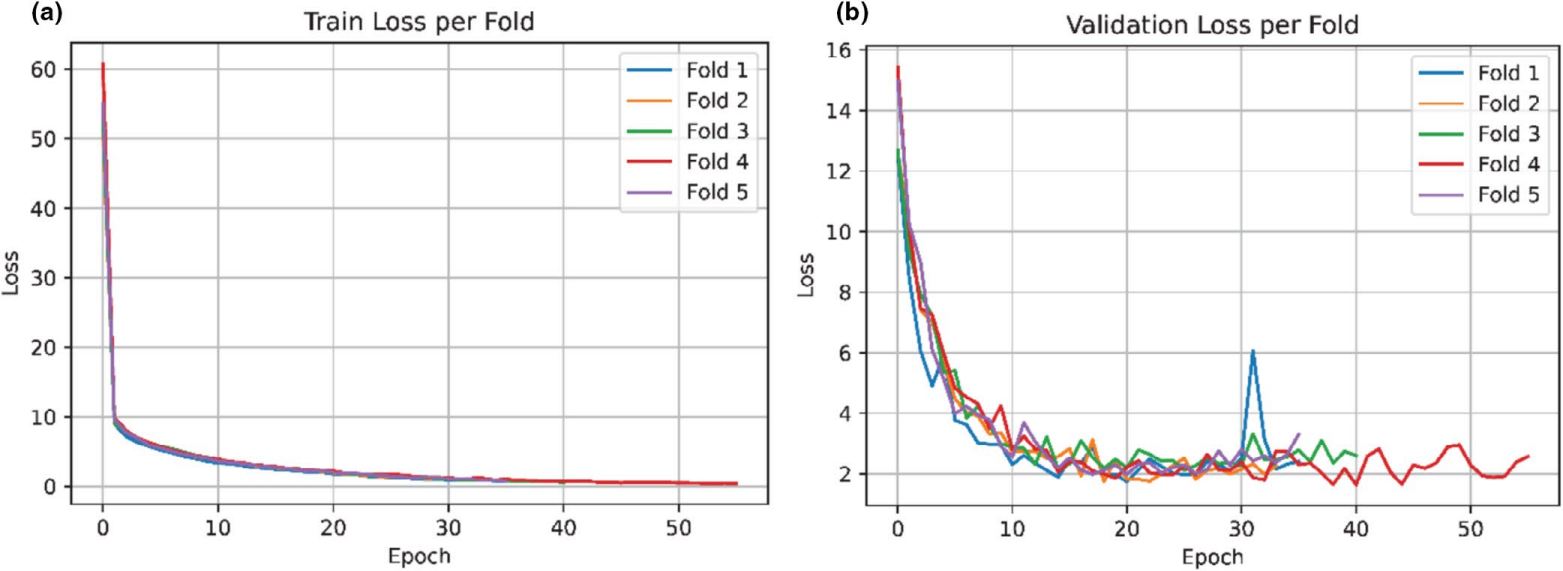
Loss curves for the non‐overlapping monitoring dataset after incorporating the single‐class dataset: (a) Training loss and (b) validation loss across fivefold.

Figures 11 and 12 illustrate the training and validation loss curves for the non‐overlapping dataset before and after the inclusion of the single‐class dataset, respectively. Table 5 reports the per‐fold macro and micro F‐scores. After incorporating the single‐class dataset, both training and validation losses converged more rapidly and reached consistently lower values across training iterations, indicating more effective and stable learning behavior. In addition, the limited variability in loss curves across folds suggests that the model exhibits consistent performance and robustness despite differences in data partitioning. This observation is further supported by the per‐fold F‐scores, which exhibit low variance across folds.

**TABLE 5:** Per‐fold validation macro and micro F‐Scores on the non‐overlapping monitoring dataset, with the single‐class dataset additionally included during training.

Fold	Macro F‐Score	Micro F‐score
Fold 1	0.968	0.984
Fold 2	0.978	0.983
Fold 3	0.969	0.972
Fold 4	0.981	0.989
Fold 5	0.945	0.957

**FIGURE 13:**
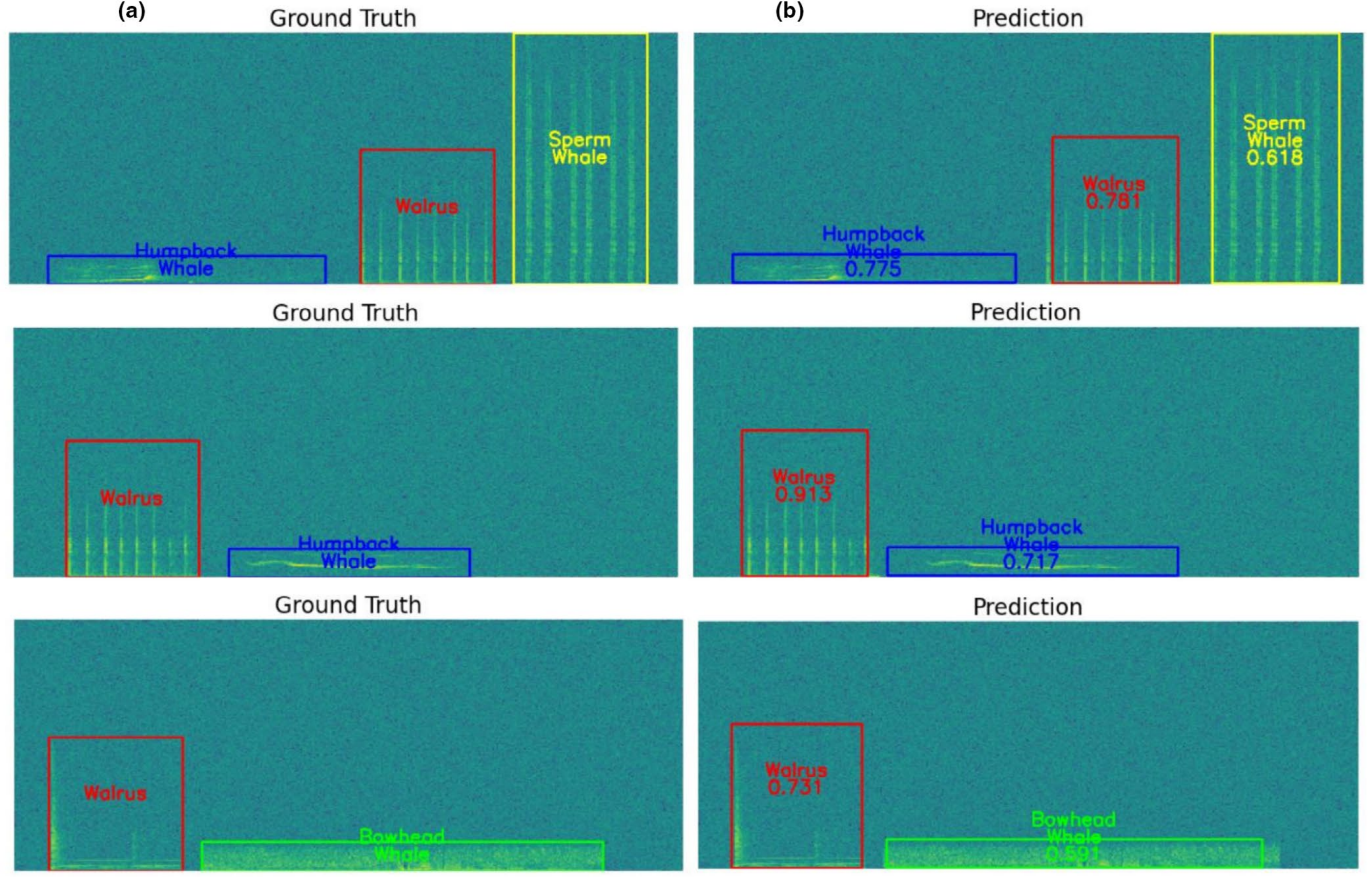
Illustrative examples comparing (a) ground‐truth annotations and (b) model predictions for the non‐overlapping monitoring dataset when the single‐class dataset was included during training. Each species is represented by a distinct color to visually distinguish different vocalization classes. Predicted events are shown as colored bounding boxes, and the numeric values displayed below each predicted class label indicate the corresponding confidence scores output by the model.

Overall, the model demonstrated strong performance for the detection and classification of multi‐species vocalizations under non‐overlapping and acoustically simplified conditions. These results establish a solid baseline for subsequent evaluations under more complex overlapping acoustic conditions. Figure 13 presents illustrative example results from the non‐overlapping dataset, demonstrating how the proposed model localizes and classifies vocalization events in spectrograms.

### 5.2 Model Assessment on Overlapping Dataset

Table 6 presents the performance metrics obtained when the model was trained and evaluated on the overlapping monitoring dataset. The single‐class dataset was included during training to enhance feature extraction and improve discrimination between vocalizations of different species. Compared with the results from the non‐overlapping dataset, an overall decline in performance was observed, which is expected given the increased acoustic complexity introduced by overlapping signals. Overlapping events create significant interference, resulting in masking and partial distortion of important signal features.

**TABLE 6:** Performance metrics when model is trained and evaluated on the overlapping monitoring dataset, with the single‐class dataset additionally included during training.

Species	Precision	Recall	F‐score
Bowhead whale	0.935	0.730	0.820
Humpback whale	0.933	0.863	0.897
Walrus	0.897	0.927	0.912
Sperm whale	0.980	0.978	0.979

This decline is most evident in the recall values, which decreased across all species, indicating a higher rate of missed detections under overlapping conditions. The effect is especially clear for the Bowhead whale and Humpback whale calls. Because the vocalizations of these species occupy similar regions within the spectrogram, the results suggest that overlapping signals within comparable regions pose significant challenges for reliable detection, even when the call patterns themselves are distinct. In contrast, Walrus and Sperm whale calls maintain relatively high recall values, likely due to their more distinctive structures, making them less susceptible to masking effects.

Despite the reduction in recall, precision values remained consistently high across all species, indicating that when detections were made, the model continued to assign correct species labels with high confidence. This suggests that the model's classification capability remained robust under overlapping conditions. F‐scores further reflect this trend, showing moderate declines for species strongly affected by overlap while remaining high for species that are less affected.

**FIGURE 14:**
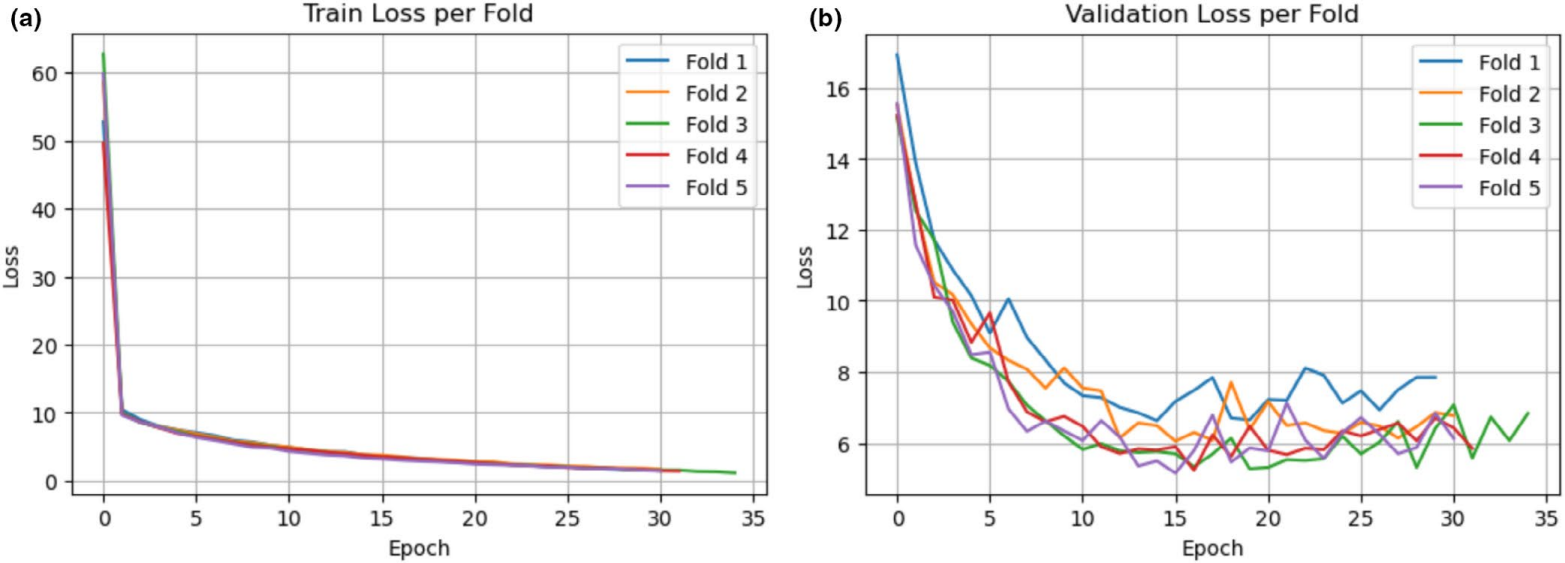
Loss curves for the overlapping monitoring dataset after incorporating the single‐class dataset: (a) Training loss and (b) validation loss across fivefold.

Figure 14 presents the training and validation loss curves across each fold. Table 7 reports the corresponding per‐fold micro and macro F‐scores. Compared with the non‐overlapping dataset, the validation loss for the overlapping dataset showed a tendency to increase after an initial decrease, indicating signs of overfitting, which is commonly observed in deep learning models trained on more complex datasets. In addition, greater variability in loss curves was observed across different folds, suggesting reduced stability during training. However, the per‐fold F‐scores show relatively limited variability, indicating that despite fluctuations during the training process, the final predictive performance remains consistent across folds. These observations highlight the importance of the ensemble approach, which helps mitigate variability and provides a more robust estimate of model performance.

**TABLE 7:** Per‐fold validation macro and micro F‐scores on the overlapping monitoring dataset, with the single‐class dataset additionally included during training.

Fold	Macro F‐score	Micro F‐score
Fold 1	0.919	0.924
Fold 2	0.920	0.930
Fold 3	0.958	0.953
Fold 4	0.936	0.950
Fold 5	0.956	0.939

**FIGURE 15:**
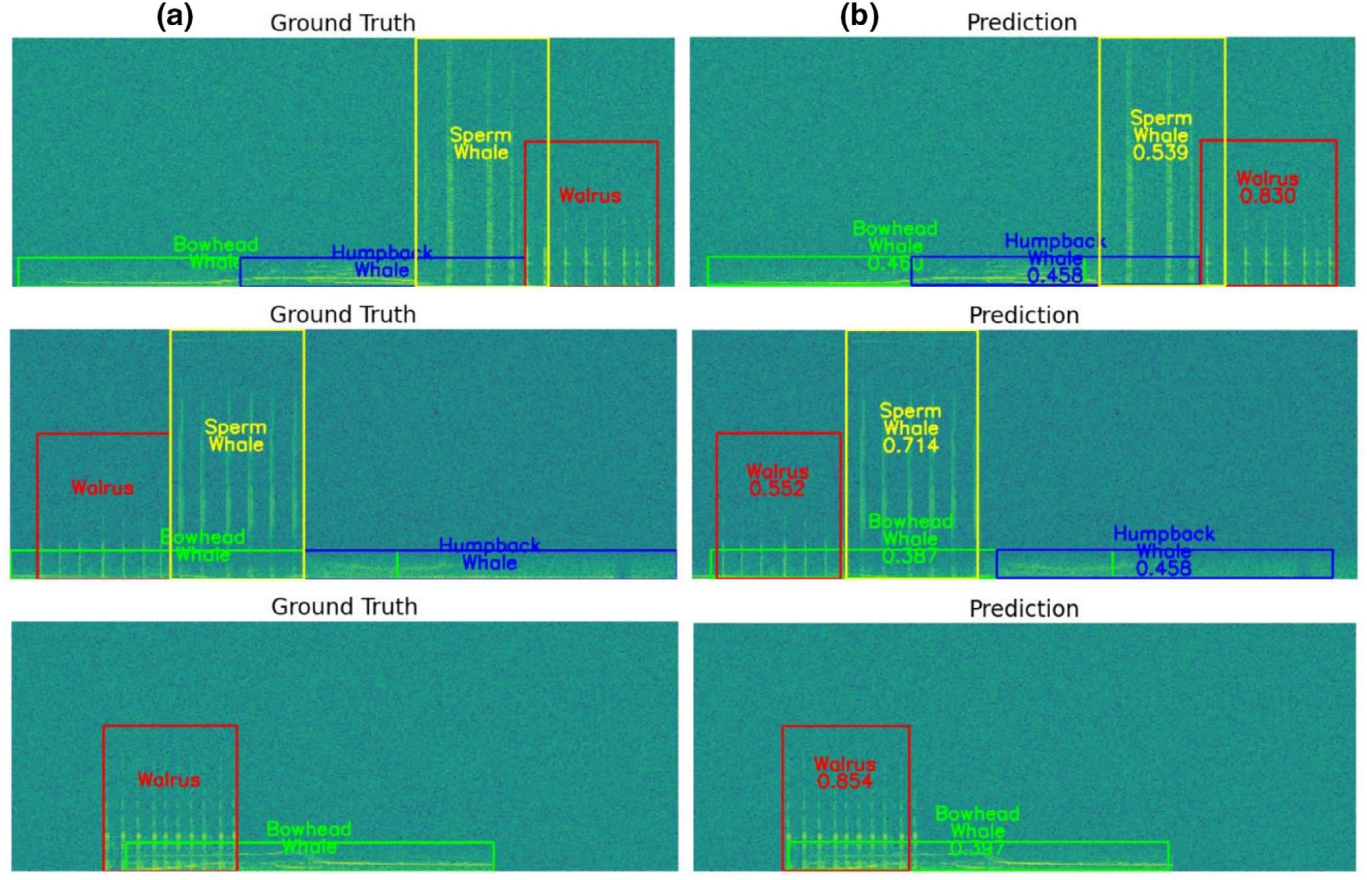
Illustrative examples comparing (a) ground‐truth annotations and (b) model predictions for the overlapping monitoring dataset when the single‐class dataset was included during training. Each species is represented by a distinct color to visually distinguish different vocalization classes. Predicted events are shown as colored bounding boxes, and the numeric values displayed below each predicted class label indicate the corresponding confidence scores output by the model.

Figure 15 presents illustrative example results comparing ground truth annotations with the model's predicted outputs for selected samples from the overlapping dataset. Despite the challenges posed by overlapping events, the model was able to identify multiple coexisting vocalizations. Overall, our findings demonstrate the model's capability of detecting and classifying multiple marine species vocalizations even under complex conditions.

#### 5.2.1 Effect of Confidence Threshold on Model Performance

The choice of confidence threshold plays a critical role in detection performance, particularly under overlapping conditions. Higher threshold values may suppress valid detections, whereas lower threshold values can result in overdetection. Therefore, selecting an appropriate threshold requires balancing precision and recall depending on the intended application.

Tables 8 and 9 present the performance metrics at confidence thresholds of 0.2 and 0.4, respectively. When the threshold is reduced to 0.2, recall increases across all species, indicating that more signals are detected. However, this is accompanied by a decrease in precision, as overdetection leads to increased misclassification, including multiple detections of the same event and false detections in background noise regions. Conversely, increasing the threshold to 0.4 results in higher precision but lower recall. In this case, fewer signals are detected, allowing the model to classify detections more accurately, but at the cost of missing some valid events.

**TABLE 8:** Performance metrics for the overlapping monitoring dataset at a confidence threshold of 0.2, with the single‐class dataset additionally included during training.

Species	Precision	Recall	F‐score
Bowhead whale	0.848	0.805	0.826
Humpback whale	0.756	0.947	0.841
Walrus	0.809	0.947	0.872
sperm whale	0.968	0.978	0.973

**TABLE 9:** Performance metrics for the overlapping monitoring dataset at a confidence threshold of 0.4, with the single‐class dataset additionally included during training.

Species	Precision	Recall	F‐score
Bowhead whale	0.939	0.433	0.592
Humpback whale	0.987	0.752	0.853
Walrus	0.926	0.913	0.919
Sperm whale	0.992	0.969	0.980

Based on these observations, the confidence threshold of 0.3 provides the most favorable balance between precision and recall in our experiments, as evidenced by the highest overall F‐scores compared to thresholds of 0.2 and 0.4. However, this choice is specific to the current dataset, and the optimal threshold may need to be re‐evaluated for different datasets, particularly those involving additional species or more complex acoustic settings.

#### 5.2.2 Performance Under Increasing Overlap Density

**FIGURE 16:**
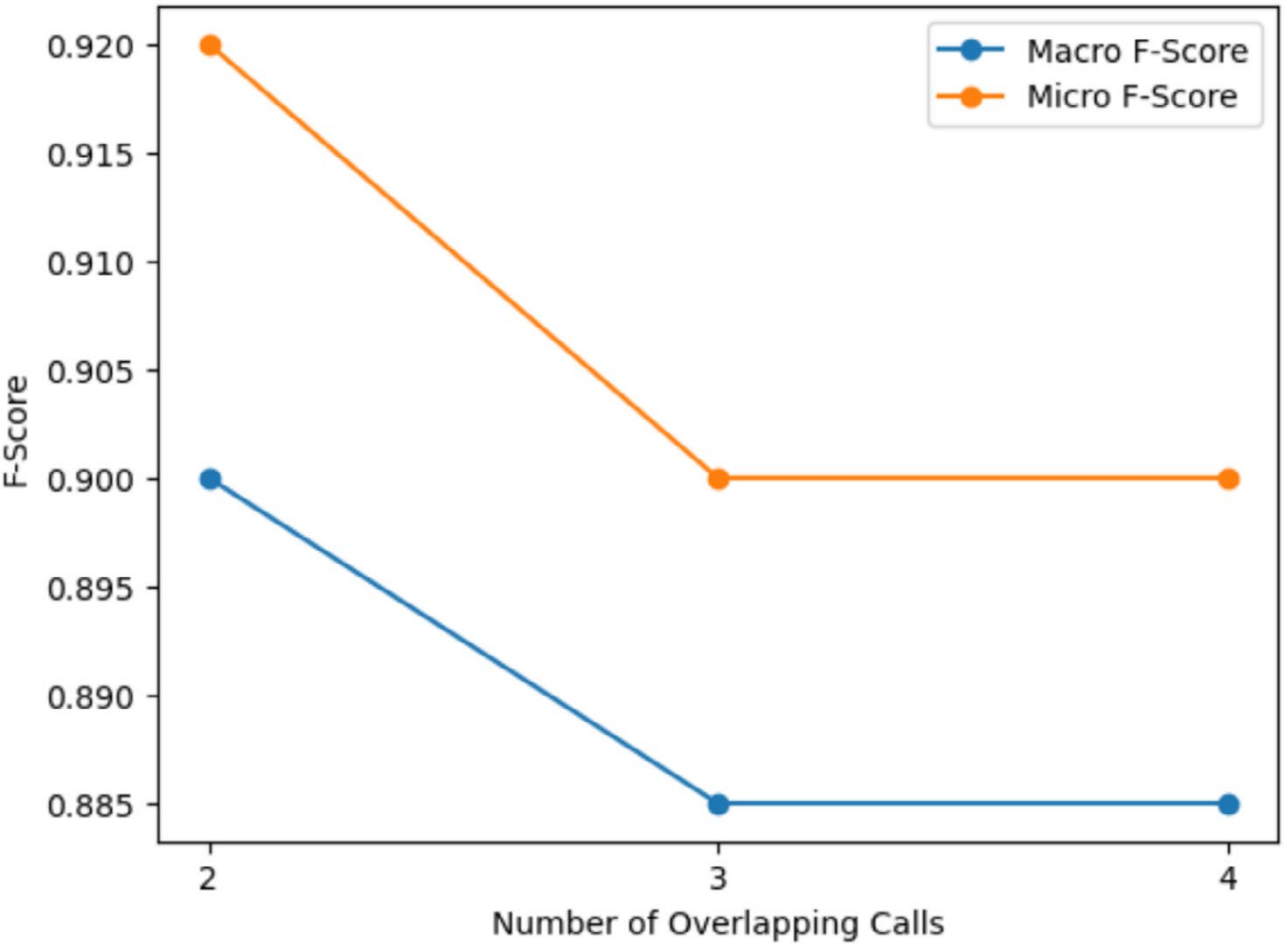
Macro and micro‐averaged F‐scores as functions of the maximum number of simultaneously overlapping signals. Results are shown for configurations allowing up to two, three, and four overlapping vocalizations.

To further examine the effect of overlap density on detection and classification performance, the original overlap configuration was systematically extended. In the baseline setup, a constraint was enforced such that no more than two signals could overlap at any given time. To evaluate the influence of increased acoustic complexity, this constraint was relaxed to allow up to three or four simultaneously overlapping signals. Model performance was then measured under these progressively denser overlap conditions. Figure 16 presents the macro and micro‐averaged F‐scores as functions of the maximum number of overlapping signals.

When overlap density increases from two to three simultaneous signals, both macro and micro F‐scores decline. The macro F‐score decreases from 0.90 to 0.885, while the micro F‐score decreases from 0.92 to 0.90. This reduction indicates that increased temporal and spectral interference negatively affects both class‐balanced performance and overall detection accuracy. This decrease suggests that certain classes may be more sensitive to overlap density, particularly those with spectrally similar vocalizations.

These results indicate that the proposed framework maintains reasonable robustness under moderate increases in overlap density. However, they should be interpreted with caution. The synthetic dataset generation process relies on random temporal placement of signals within each configuration. As a result, although the total number of training and test samples is identical across overlap conditions, their specific composition differs at each level. Consequently, performance variations may be affected by stochastic differences of the datasets rather than purely systematic effects of overlap density. Nevertheless, the observed overall trend of decreasing performance with increasing overlap is consistent with theoretical expectations, as higher overlap density increases distortion within time‐frequency representations.

### 5.3 Limitations and Future Works

Although the present study demonstrates the potential of a YOLO‐inspired framework for marine mammal vocalization detection and classification, several limitations remain that can influence its generalization to real‐world monitoring applications. First, model training and evaluation were conducted entirely on synthetically generated monitoring datasets. While these datasets provided a practical and controlled solution due to the limited availability of continuous recordings, they cannot fully replicate the complexity of natural marine soundscapes. Furthermore, although the training and test sets are constructed from separate signal segments obtained during the initial train–test split, both datasets are generated using the same synthetic pipeline, including identical SNR ranges, noise models, and overlap statistics. As a result, the test set is not fully independent and represents an in‐distribution evaluation that shares the same generative assumptions as the training data.

Specifically, key acoustic processes were not explicitly modeled. Real marine environments involve frequency‐dependent attenuation, multipath propagation, and refraction, all of which can distort signal structure and reduce SNR with increasing distance. Background soundscapes in nature are also highly variable, including diverse biological and anthropogenic noise, whereas synthetic mixing procedures represent only a limited subset of such variability. As a result, performance metrics obtained under synthetic conditions may represent an optimistic estimate.

Second, although the automated annotation procedure was revised to operate directly on raw spectrogram matrices, it remains dependent on amplitude‐based thresholding and species‐level frequency assumptions. While approximate frequency ranges for many marine mammal vocalizations are often known from prior bioacoustic studies, natural recordings can exhibit greater variability in call structure and spectral distribution. In particular, under complex, overlapping conditions, the use of averaged or representative frequency boundaries may introduce uncertainty in bounding box definition. Although this approach was appropriate for the curated synthetic datasets used in this study, its robustness under highly variable real‐world acoustic conditions requires further investigation. Additionally, because the method relies on amplitude‐based thresholding, the optimal threshold is likely to depend on signal‐to‐noise conditions and dataset‐specific characteristics. As such, recalibration may be necessary when applying this method to datasets with different noise levels or variability.

Finally, the present work is intended as a methodological proof‐of‐concept rather than a fully optimized real‐time deployment system. In its current configuration, the model contains approximately 7.05 million trainable parameters and achieves an average inference time of approximately 110 milliseconds per five‐second spectrogram segment on a single NVIDIA RTX 3090 GPU. While these results indicate reasonable computational feasibility, further exploration of model compression techniques and hardware‐efficient implementations will be necessary to support practical large‐scale or real‐time field deployment.

Furthermore, developing a systematic framework for analyzing detection errors will be important for future applications. In ecological monitoring contexts, false negatives may lead to underestimation of species presence and vocal activity, potentially biasing conclusions regarding population distribution. Conversely, false positives may overestimate species activity or co‐occurrence, which could mislead interpretations of species presence and interactions. A clearer characterization of when and why these errors occur will therefore be essential to ensure that automated detection outputs are interpreted appropriately in ecological analyses.

## 6 Conclusion

In this study, we proposed a deep learning‐based framework for the detection and classification of marine species vocalizations using spectrogram representations derived from acoustic monitoring data. The proposed network integrates preprocessing methods such as the Short‐Time Fourier Transform, a modified CutMix technique adapted to spectrogram characteristics, and a modified one‐dimensional detection model inspired by the YOLO architecture. This design enables the model to effectively identify and classify vocalization events within a given time window.

To address the challenge of limited raw acoustic recordings, the available data were manually segmented and combined to construct multi‐signal datasets representing both non‐overlapping and overlapping monitoring conditions. The non‐overlapping dataset provided a strong baseline for evaluating model performance under ideal conditions, while the overlapping dataset introduced greater complexity, simulating more realistic acoustic environments. However, such datasets require detailed annotations to specify each vocalization event within the spectrogram, typically a time‐consuming manual process. This study incorporated an automated annotation method, enabling faster and more consistent spectrogram annotation and allowing a greater volume of training data to be constructed.

Despite these advances, certain limitations remain. While the synthetic datasets used in this study provided a practical solution in the absence of continuous monitoring data, they cannot fully replicate the complexity of real continuous monitoring recordings. Environmental noise, acoustic variability, and other unpredictable characteristics of natural soundscapes are difficult to simulate accurately. Therefore, the results presented in this study should be regarded as a foundational baseline rather than a direct indicator of real‐world performance. Similarly, the proposed automated annotation method might be effective under controlled conditions, but may not generalize directly to real‐world recordings, where acoustic diversity, higher noise levels, and more complex, overlapping sources are common.

Future research should therefore focus on extending this framework to long‐duration acoustic monitoring datasets. Incorporating such data will enable the model to learn more complex acoustic patterns and improve its generalizability. Additional enhancements, such as integrating unsupervised learning techniques, may further improve model transferability. By learning generalized acoustic representations from unlabeled recordings, the framework could be adapted more effectively to new monitoring sites, environmental conditions, and potentially previously undocumented vocalization patterns. Beyond technical improvements, such developments could enable broader ecological applications, including the study of multi‐species co‐occurrence patterns and temporal dynamics of vocal activity. With continued development, the proposed approach has the potential to support efficient ecological monitoring that can significantly enhance marine ecosystem management and conservation efforts.

## Author Contributions

**Min Jun Kim:** conceptualization (lead), methodology (lead), software (lead), visualization (lead), writing – original draft (lead), writing – review and editing (equal). **Juan Lee:** conceptualization (equal), visualization (equal), writing – review and editing (equal). **Yongchae Cho:** supervision (supporting), writing – review and editing (equal). **Won‐Ki Kim:** supervision (lead), writing – review and editing (supporting). **Jungyong Park:** resources (equal), supervision (supporting), writing – review and editing (supporting). **Dawoon Lee:** resources (supporting), supervision (supporting). **Ho Seuk Bae:** funding acquisition (lead), project administration (lead), resources (equal), supervision (supporting).

## Funding

This work was supported by Agency for Defense Development, UE240509DD.

## Conflicts of Interest

The authors declare no conflicts of interest.

## Data Availability

The raw acoustic recordings used in this study are publicly available from the Watkins Marine Mammal Sound Database, Woods Hole Oceanographic Institution. The dataset can be accessed at: https://www.whoi.edu/. The code developed for this study has been made publicly available at: https://github.com/mjk9898/YOLO‐Inspired_MarineSpeciesDetection.git.
